# PaddyVarietyBD: Classifying paddy variations of Bangladesh with a novel image dataset

**DOI:** 10.1016/j.dib.2025.111514

**Published:** 2025-03-23

**Authors:** Md Tahsin, Muhammad Ibrahim, Anika Tabassum Nafisa, Maksura Binte Rabbani Nuha, Mehrab Islam Arnab, Md. Hasanul Ferdaus, Mohammad Manzurul Islam, Mohammad Rifat Ahmmad Rashid, Taskeed Jabid, Md. Sawkat Ali, Nishat Tasnim Niloy

**Affiliations:** a Department of Computer Science and Engineering, East West University, Aftabnagar, Dhaka, Bangladesh; b Department of Computer Science and Engineering, University of Dhaka, Dhaka 1000, Bangladesh; c Department of Computer Science and Engineering, International Islamic University Chittagong, Chittagong, Bangladesh

**Keywords:** Agriculture, Microscopic image, Paddy variety, Image classification, Artificial intelligence, Computer vision

## Abstract

Among countless crop varieties produced worldwide, the staple food of most of Asia, some parts of Europe, and North America is rice. Being an essential food item, rice offers an integral contribution to the economy of countries like China, India, Bangladesh, Pakistan, Indonesia, and so on. Scientists have long been working on developing new and improved rice species to battle different environmental hindrances and natural calamities. Although numerous research and studies have been conducted on this diverse crop, artificial intelligence, in particular, machine learning has not been practiced in this field with its full potential. The key factors behind this lag include the unavailability of standard and ready-to-use datasets. Intending to mitigate this drawback, this paper proposes an image dataset of paddy species to assist researchers and scientists in classifying, analyzing, and evaluating paddy classes. To the best of our knowledge, this is the first standard and open dataset of paddy varieties in Bangladesh. The rice sample was collected from two places namely – Bangladesh Institute of Nuclear Agriculture (BINA) and the Bangladesh Institute of Rice Research Institute (BRRI) where agrarian scientists work on developing new or improving existing paddy species. The dataset contains 14,000 RGB microscopic images of each paddy kernel. The enormity and inclusivity of the dataset make it useful for global research purposes. The dataset can be a useful resource not only in the area of artificial intelligence, but also in agriculture, botanical, and economic research.

Specifications Table

**Table:** 

Subject	Agronomy and Crop Science
Specific subject area	Classification of paddy kernel variety
Type of data	Analyzed, curated, and filtered.
Data collection	35 unique paddy varieties invented in Bangladesh have been selected for this study. For this purpose, the paddy kernels were collected from two different Agricultural Research Centres in Bangladesh. Upon collection of the rice kernels, images of 100 paddy kernels from each variety were captured in 4 different orientations.
Data source location	1. Bangladesh Institute of Nuclear Agriculture (BINA) 2. angladesh Institute of Rice Research Institute (BRRI)
Data accessibility	Repository name: Mendeley Data Data identification number: 10.17632/tx277syzhm.1 Direct URL to data: https://data.mendeley.com/datasets/tx277syzhm/1 Researchers may access the URL and find the original and augmented image folders. They can directly download the image to conduct their research work.
Related research article	None*.*

## 1 Value of the Data

• Being one of the top paddies producing and consuming countries worldwide, an image dataset consisting of microscopic RGB images of paddy varieties has been developed. These varieties have their distinct planting season for each variation, optimal geographical location for cultivation, production rate as well and morphological features such as size, shape, and color.
• The dataset consists of 35 unique species of paddy that are produced in Bangladesh and are unexplored in the field of artificial intelligence. This dataset contains 400 microscopic colored pictures from each variety, thereby totaling 14,000 original images altogether. Images were taken with two microscopic camera devices having 1000x and 1600x zooming, respectively.
• The curated dataset can be used for paddy variety classification and analysis of further characteristic differences between the classes. The massive quantity and diversity of these data are expected to be beneficial for further research in agriculture. Moreover, the dataset may be useful in other research fields including biotechnology, phytopathology, and crop science.
• In the sustainable agricultural research domain, artificial intelligence and machine learning are not yet harnessed to their full potential. Automated technologies can bring revolutionary changes in this field. Tedious research with crops can be prone to errors and these manual processes require much resource and manpower. Machine-driven systems can be precise, meticulous, and time-saving. Nevertheless, this process is often hindered by limited resources inadequacy, and unavailability of properly structured and standard datasets. To minimize this gap in the existing literature, this research develops a standard dataset explicitly designed for classifying paddy varieties for the artificial intelligence models [1]

## 2 Background

Rice is a crop that enhances the country's economy by bringing foreign exchange. Exporting rice varieties overseas has been an age-long practice in the South-Asian subcontinent. BD39, BD87, and Binadhan7 are varieties that have high export rates. The kernels are thinner and longer than other varieties available in the market making it more demandable internationally. Likewise, some rice types are also fragrant which adds to their asset value. Fragrant rice is used for various purposes in cooking all around the world.

The compiled data includes two such varieties with aromatic notes named BD70 and BD75. Varieties named Binadhan11, Binadhan14, Binadhan16, Binadhan17, Binadhan23, Binadhan24, Binadhan25, and Binadhan26 provide a high yield that is profitable for the farmers and the agronomy as a whole. In this dataset, there are 20 unique varieties of “AmonDhan” 4 varieties of “BoroDhan” and other derivatives of “AushDhan”. Some of these varieties are developed by agricultural research institutes in Bangladesh to combat adverse environments in different geographical locations. For instance, floods and drought are the most common calamities in Bangladesh that highly affect the agricultural economy. BD52, BD79, BD91, BR22, BR23, Binadhan11, and Binadhan12 are varieties that are flood resilient and can survive up to 18-21 days being submerged in water. These varieties are beneficial for farming in lands that are prone to flood. On the contrary, BD33, BD56, BD57, BD95, and Binadhan17 are drought-tolerant variations of Amon. The absence of rain for up to 10-12 days does not damage the reproduction cycle for these species. Also, species like Binadhan8 and Binadhan10 are salt tolerant and provide high yields in even higher salinity levels [2].

Some of the sensitive varieties require optimal water conditions to achieve the expected yield. BD72, BD87, and BD93 are such varieties that require ideal watered soil for proper production. On the contrary, some varieties are resilient to low tide. Low tide-resistant varieties included in the dataset are BR23 and BD76. Light sensitivity is also a common phenomenon in rice plants [10]. BR22, BR23, BD30, Binadhan8, Binadhan10, Binadhan17, Binadhan23, Binadhan24, and Binahan25 are some light-sensitive varieties that provide the highest yield on the post-flood timeline [11]. On the contrary, Binadhan14 is a high-temperature tolerant derivative of BoroDhan with a high yield. Binadhan19 is such a variety that is mainly developed to be cultivated in hilly regions with higher throughput. Besides that, one insect-tolerant species is Binadhan20. This species has reddish long-slender rice grains [2,9].

## 3 Data Description

The paddy varieties have been collected from two agricultural research institutes in Bangladesh. Primarily, 20 varieties were collected from Bangladesh Rice Research Institute (BRRI), Gazipur. Afterward, another 15 varieties from the Bangladesh Institute of Nuclear Agriculture (BINA), thereby totaling 35 varieties were collected [2].

In the next step, 400 RGB microscopic images of each paddy variety have been stored in distinct folders that are labeled by the name of the respective paddy species. There are 35 separate subfolders in the dataset – one for each paddy variation. The subfolders are then placed in a main folder named “Paddy Collection”. Each picture has a dimension of 640×480 pixels, and the images are in JPG format.

Every class included in the dataset has distinguished features to be classifiable by artificial intelligence models. Signature attributes of each paddy variety are focused while capturing the photos. In Table 1, representative images from each of the 35 classes are shown keeping their respective unique features, namely color, length, and width in focus.

**Table 1:** Sample images and characteristics of 35 paddy varieties used in this dataset.

Paddy Variety	Characteristics	Sample Image
BR22	The plant has a height of 125 cm. It features paddy, which is clean, short, bold, and translucent. It exhibits resistance to tungro and sheath blight, with moderate tolerance to the yellow stem borer.	
BD33	The full-grown plant is 100 cm tall. The paddy is short in length and dense in characteristics. The plant remains upright and sturdy. The plant leaves are somewhat wider than the typical varieties and 2–3 cm beneath the leaf tip, curly folds appear. Along the folds, the paddy displays a light brown color and the breed's lifespan is 118 days.	
BD30	The paddy is medium-thin and white with photosensitivity. Upon reaching full growth, the plant stands at 120 cm. The leaves rise progressively till the end of their life span, which is 145 days.	
BD85	The height of the plant is 106 cm. The paddy is long and thin. The stem is strong, so it does not sag. The leaves are slightly wider and steeper than other common varieties. The life span of this breed is 120 days.	
BD56	The height of a mature plant is 115 cm. At the growing stage, the plant is taller than BR11 in both size and shape. Leaves are dark green with erect and long variety. The ripe paddy signifies a reddish color. Paddy grain color resembles the golden kind, with thicker and longer characteristics. A thousand nutritious paddy grains weigh about 23.6 g. Without flood, the plant's lifespan is 135 days which increases to 160 days if it endures flood for nearly a month.	
BD93	The Ufshi paddy displays features of the BRRI Dhan49 variety with similarities in growth, size, and shape. The leaves are dark green in color and upright. Mature plants are 117 cm tall. The amylose level is 26.1 % and protein content is 7.5 %. A 1000 paddy weighs 18.95 g. The paddy grains are reddish, with medium coarse and white rice. This breed has a lifespan of 134 days.	
BD91	Characteristics of this breed are moderate elongation, submergence tolerance, and cultivation suitability in pre-shallow waterlogged areas. The seedlings of this breed are tall and grow quickly, reaching 190 cm, with strong bases that tolerate leaning. It has the characteristics of Ufshi rice, such as erect, long, well-spread roots and dark green leaves. The stem has vascular bundles and air passages that are 3 times larger than those of conventional varieties. The perennial stem remains strong and green after the paddy ripens and can be propagated using stem cuttings. These strong stems are also appropriate for sorghum crops. The grains are light brown and medium-thick, with numerous nutritious grains that do not fall easily. The rice is medium coarse, white, and crisp. A thousand nutritious grains weigh approximately 26.0 g. This variety is not sensitive to light, and it survives for 152–156 days.	
BD49	The tree grows to 100 cm tall. Paddy is slimmer than BR11 and BRRI Dhan32. Its rice is slightly coarse and white. The protein percentage in the rice is 8.5 %. This type survives for 135 days.	
BD51	A variety that can withstand flash floods and have low light sensitivity. And the stem's rigidity stops it from bending. As the stem is strong, it does not bend. The paddy is medium-thin, white, and transparent. The plant's height is 90 cm. The life span of this breed is 140–145 days.	
BD52	The paddy variety of Aman has flood tolerance and low light sensitivity. Reaching an impressive mature height of 116 cm with a strong stem base. Paddy is medium coarse and waterlogging tolerant. Life span is 140–145 days in a normal flood-free environment and 155–160 days in a 14-day flash flood.	
BD76	This variety of paddy contains all the characteristics of modern Ufshi paddy. In favorable conditions, the plants are tall, with an average mature height of 140 cm, and robust. The stems are strong and resistant to falling, while the leaves are erect and green. The grains are nutrient-rich, with 1,000 grains weighing approximately 25.6 g. The lifespan of this variety is around 153 days.	
BD95	This variety exhibits all the traits of modern Ufshi paddy. The mature plant of this breed reaches an average height of 120 cm. It resembles the BRRI Dhan 49 variety during the growth stage, size, and shape. The leaves are erect and dark green. The paddy is medium-thick and white, with grains that are dark red. The weight of 1,000 grains is approximately 21.50 g. The paddy contains 28.0 % amylose and 8.0 % protein. The lifespan of this variety is 125 days.	
BD57	The size and shape of the plant at the growing stage are slightly taller than BR11. The height of a fully grown plant is 110–115 cm. The leaves of this variety are erect and long and the color of the leaves is pale green. The main feature of this variety is that the grain of the paddy is thin, and the tip is very straight. 1000 nutritious rice weighs about 19.2 g. The color of the ripe paddy is like straw. The life span of this breed is 100–105 days.	
BD87	The full-grown tree is 122 cm tall. The sturdy stem prevents it from drooping even when it grows tall. The leaves are light green, erect, and longer and wider than BRRI49. During ripening, the stems and leaves remain green. Grains are long and thin. The life span of this breed is 125–128 days. And a notable fact is that 1000 nutritious rice weighs about 24.1 g. Amylose is 27 % in long and thin rice.	
BD70	Aman is the most productive variety of fragrant paddy. The plant exhibits erect, tall leaves and reaches a height of 125 cm. The color of paddy grains is like straw, very long, thin, and aromatic. The tip of the grain has a small spike and a colored tip. The shape of the paddy is quite long and thin and the color is white. The weight of 1,000 grains of this nutritious rice is about 20 g. The amount of amylose in rice is 21.7 %. This variety has a growth cycle of 130 days.	
BR23	The tree reaches a height of 105 cm. The paddy is clean, long, slender, and white. Moderately tolerant to sheath blight and white-backed plant hopper and it exhibits tolerance to blast, salinity, and waterlogged conditions (to some extent).	
BD72	This high-yielding Aman paddy variety is rich in zinc. The height of the plant is 116 cm tall and is strong and does not fall. The leaves are broad and dark green. The grain at the head of the paddy grain has small spikes. The shape of the paddy is long, thick, and white. An intriguing detail is that a thousand grains of this nutritious rice weigh approximately 27.9 g. The paddy contains 26.0 % amylose and has a lifespan of 125–130 days.	
BD79	All the characteristics of modern Ufshi paddy are present there. The size and shape of the plant at the growing stage are almost like that of BRRI Dhan49, but the grain is slightly longer and thicker than BRRI Dhan49. The leaves are erect and long and almost green when they mature. The height of a fully grown tree is 112 cm. Amylose in paddy is 25.2 % and protein is 7.8 %. 1000 nutritious rice weighs 22.6 g.	
BD75	This variety comprises all the modern Ufshi paddy features. The height of a fully grown plant is 101–110 cm. The stem is strong, so it does not bend, and the paddy does not fall from the ear. The leaves are erect, broad, and long and the color of the leaves is dark green. The color of rice grains is golden and medium-thin. The weight of 1000 nutritious rice is about 21 g. The life span of this variety is 110–115 days.	
BD34	This variety is aromatic and small like Kalijira paddy and is very useful for making Pulao. The fully grown tree reaches a height of 117 cm and has low light sensitivity.	
Binadhan7	This variety of Aman is known for its high yield and high-quality seedlings. It features dark green and wide leaves. The tree is thick and hard and does not fall. Life span 110–120 days. The color of this paddy is bright. Both rice and paddy are long and thin, delicious to eat, and which is why the market value is high and suitable for export. Also, the amount of amylose in rice is 24–25 % [12].	
Binadhan8	This Boro variety is characterized by its salt tolerance, high yield, and improved quality. Tolerant to salinity levels of 8–10 ds/m from bud stage to maturity and 12–14 ds/m at seedling stage. It has a lifespan of 130–135 days.	
Binadhan10	This paddy variety of “Boro” is light sensitive, salt tolerant, high, and yielding with improved quality. Tolerant to salinity levels of 10–12 ds/m from bud stage to maturity and 12–14 ds/m at seedling stage with a lifespan of 125–130 days.	
Binadhan11	This Aman rice variety is flood-tolerant and high-yielding and can fully submerge in water for up to 25 days. Life span 110–115 days. Flood-tolerant varieties are suitable for cultivation in both flash flood-prone and flood-free areas of the country during the Aman season.	
Binadhan12	It is a paddy variety of Aman which is flood-tolerant and high-yielding and can survive fully submerged in water for up to 25 days. Life span 125–130 days. Flood-tolerant varieties are suitable for cultivation in both flash flood-prone and flood-free areas of the country during the Aman season.	
Binadhan14	This Boro paddy variety has high-yielding and high-temperature tolerance. Life span 120–130 days.	
Binadhan16	An Aman paddy variety with high-yielding and short-lived. Life span 100–105 days. Paddy does not fall. Its rice is long and thin.	
Binadhan17	Aman paddy variety with less fertilizer, light insensitive, and high qualities. Drought tolerant (30 % less water required), short-lived (lifespan 112–118 days), and high yielding [13].	
Binadhan19	The cultivation of this variety is suitable in the Aush and Aman seasons. Paddy is narrow and long. Life span 95–105 days. It is suitable for direct planting in rain-dependent conditions throughout the country which includes Barendra and hilly areas. Irrigation water is beneficial. The growth of the plant stops during severe drought. But when the favorable environment comes, the plant can complete growth and give normal yield.	
Binadhan20	The paddy is red in color, long and thin. Life span 125–130 days. The brown plant is moderately resistant to grasshoppers. 26.5 ppm of zinc is present in zinc-rich paddy, and coarse rice. 20–31 ppm iron present.	
Binadhan21	The cultivation of this variety can be done in the Aush season. The height of a fully grown tree is 94–96 cm. As the trees are short and steep, they do not lean. Life span 100–105 days. 1000 paddy weighs 21.3 g. Paddy is white, long, and thin. The amount of amylose in rice is 24.9 %. This variety results in rice that cooks up a fluffy and delicious flavor [14].	
Binadhan23	These Aman variety season crops are suitable for tidal, salinity, and flood-prone areas of the country. It is short-lived, lifetime 115–125 days, high-yielding, and light insensitive. At maturity, the variety can tolerate salinity up to 8 ds/m and submergence up to 15 days. The paddy is medium-thin.	
Binadhan24	This high-yielding variety has a short duration, with a lifespan of 143–145 days. It is light-insensitive and produces long to medium fine grains. Even at maturity, the stem remains green and firm. The tree is strong and does not fall at all. A full-grown tree 95–97 cm long.	
Binadhan25	This Boro paddy variety is high-yielding, light-insensitive, and has a short duration, with a lifespan of 138–148 days. The leaves are erect, narrow, and medium, dark green. The tiller remains dark green and erect even after the paddy matures. The tree is tall but does not lean heavily. The height of a fully grown tree is 116 cm. Each plant has 10–12 buds. The average length of rhyme is 27.0 cm. long 150–155 nutritious grains per seed. The average weight of 1000 nutritious rice is 19.7 g. The amylose content of paddy grains is 25.1 %, and the protein content is 6.6 %. This variety produces neat, white, and tasty rice which brings out a high market value and is suitable for export.	
Binadhan26	A variety of Aman that contains high yielding and a lifespan of 115–120 days. Amylose content in paddy is 26.4 percent and protein content is 9.4 mg/kg. Bacterial Leaf Blight (BLB) resistance genes Xa4 and xa5 are present in the variety.	

The lifespan of paddy varieties in Bangladesh is influenced by the specific type, growing season, and environmental factors. Paddy cultivation is primarily categorized into three types: Aush, Aman, and Boro, each characterized by a unique growth cycle. Aush rice, which is cultivated in the pre-monsoon season, has a relatively short lifespan of 80 to 120 days, with sowing occurring in early spring and harvesting in the summer months. This variety requires less water compared to others. Aman rice, the most extensively grown type, is cultivated during the monsoon season and has a lifespan ranging from 130 to 150 days. It is typically sown in late spring or early summer and harvested in late autumn, utilizing both direct seeding and transplantation methods. Boro rice, which is grown in winter with the aid of irrigation, has the longest lifespan of 140 to 160 days, with sowing occurring in early winter and harvesting in spring. This variety is heavily dependent on effective water management and is recognized for its high yield. The lifespan of these varieties is affected by various factors, including climate, soil fertility, and agricultural practices. Hybrid and high-yielding varieties tend to mature more quickly, while traditional varieties require a longer growth period. Efficient management of water, fertilizers, and pests is essential for maximizing growth and yield across all paddy types [2].

Summarized information of the PaddyVarietyBD dataset is represented in Table 2. This table contains a brief description of the data type of the image file along with its format, the number of images, the number of classes that are collected, the number of images in each category, how the data was acquired, the locations from where the data was collected and the field where the researchers can use it.

**Table 2:** PaddyvarietyBD dataset information in brief.

**Type of data**	640 × 480 pixels RGB microscopic paddy images.
**Data format**	JPG
**Number of images**	400 images of each paddy class adding up to 14000 images of 35 rice varieties.
**Crop**	Paddy
**Number of classes**	35 (BD30, BD33, BD34, BD49, BD51, BD52, BD56, BD57, BD70, BD72, BD75, BD76, BD79, BD85, BD87, BD91, BD93, BD95, Binadhan7, Binadhan8, Binadhan10, Binadhan11, Binadhan12, Binadhan14, Binadhan16, Binadhan17, Binadhan19, Binadhan20, Binadhan21, Binadhan23, Binadhan24, Binadhan25, Binadhan26, BR22, BR23)
**Distribution of instances**	Each category contains 400 images.
**How data are acquired**	100 paddy kernels from each rice species were captured one at a time in 4 orientations.
**Data source locations**	**1.** Bangladesh Institute of Nuclear Agriculture (BINA) and **2.** Bangladesh Institute of Rice Research Institute (BRRI), Gazipur, Bangladesh.
**Where applicable**	Suitable to classify distinct paddy varieties.

## 4 Experimental Design, Materials and Methods

In recent years, there has been an enormous boost in the field of artificial intelligence and automation of systems. In particular, machine learning and deep learning models have influenced almost every corner of life by producing systems that are automated, hassle-free, accurate, and less prone to human error. Crucial and significant sectors of society such as agriculture, healthcare, education, economy, and many more are now influenced by artificial intelligence systems. Though the application of artificial intelligence in agriculture is still at a comparatively lower stage, the scenario is changing with the advancement of new technologies. Automatic crop classification, disease detection, and early prevention of diseases in crops are some of the widely known usages of automation in this domain [3]. The prerequisite of any machine learning algorithm is the availability of an appropriate, standard, and robust dataset. In order to achieve a fully functional, high-performance automated machine learning system, the data need to be well organized, high quality, relevant, and substantial.

Enabling automated technologies in agriculture for the advancement of paddy production and uplifting the farming community can be game-changing. However, there is a scarcity of image datasets consisting of the unique and special attributes of different kinds of rice kernels that are developed in Bangladesh by researchers [4,5].

### 4.1 Dataset Collection and Preparation

**Fig. 1:**
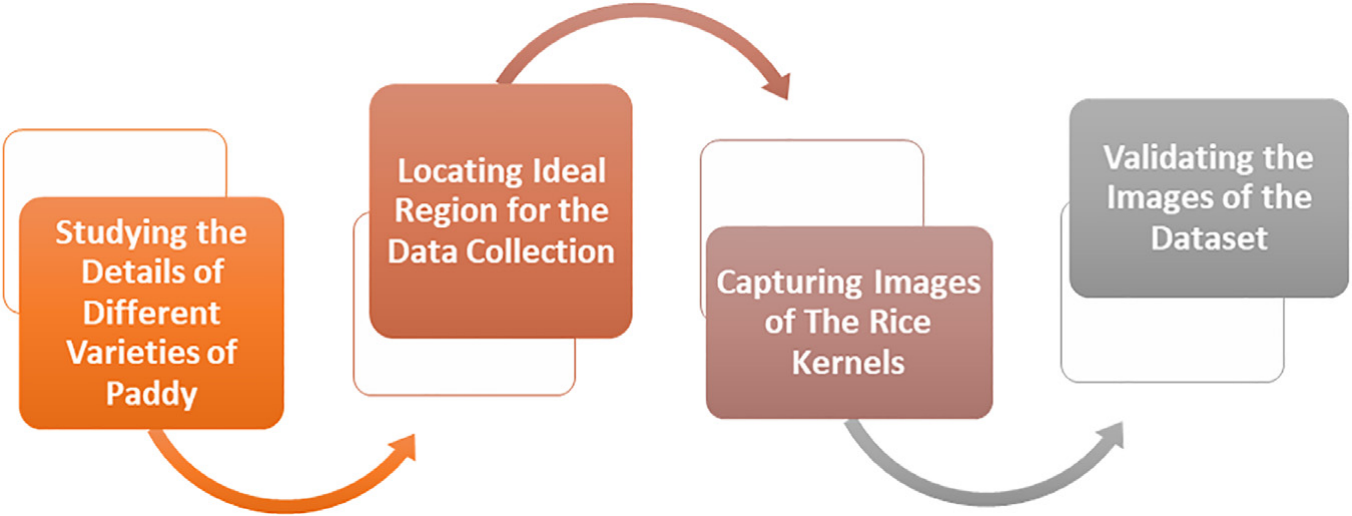
Steps of data collection and preparation.

A structured and systematic process focusing on extracting high-quality, multifaceted, and wide-ranging data has been followed. Agricultural experts were employed to meticulously label the acquired images of each rice kernel. Following that, to get the dataset in the optimal format for machine learning model training, images are converted into uniform size and shape [8]. Any unwanted background noise is excluded from the images. Fig. 1 illustrates the summary of the dataset development steps. To prepare this dataset, the followings steps have been Fundamental steps that are followed to develop this dataset are as follows:

• Acquiring key information regarding rice varieties available as well as developed in Bangladesh.
• Discovering the institutes from where the maximum number of rice classes can be gathered.
• Collecting the samples that are in ideal condition to be captured for the dataset.
• Capturing the images of kernels from the same class that are variant in color size and shape to diversify the data.
• Validating the images of the dataset by examining their quality.

### 4.2 Studying the Details of Different Varieties of Paddy

From Bangladesh's perspective of consumption and production, rice is the highest-produced and consuming crop. Farmers in Bangladesh depend on paddy production for the major part. In the past three decades, rice has been cultivated in 10.5 million hectares of land in Bangladesh which is approximately 75 % of the total cropped land [8].

Plenty of research is being conducted in Bangladesh to achieve paddy varieties with high yield, being climate-friendly and insect-resistant. Among all the varieties of paddy available in Bangladesh, 20 varieties of “Amon” have been chosen for developing this dataset. Each of these varieties is different in terms of morphological and other features. Morphological features include color, shape, and size. Other features are yield, optimal climate, and production cycle.

### 4.3 Locating the Ideal Region for the Data Collection

There are plenty of paddy fields that are dispersed throughout Bangladesh. Collecting samples directly from paddy fields or farmers or from markets could cause inconsistency among the data classes. This could lead to a scarcity of samples, transportation hassle, communication issues, and faulty labeling [6]. With an aim to avoid such issues, the sample rice kernels were collected directly from rice research institutes, namely Bangladesh Rice Research Institute (BRRI) and Bangladesh Institute of Nuclear Agriculture (BINA). Exclusive paddy varieties that are developed in Bangladesh are stored in these institutes in prime condition. Kernels in such conditions are the supreme choice for further research purposes. In this study, 35 varieties of these ideally stored kernels have been utilized to develop the dataset. Collecting from research institutes ensures accurate and expert labeling of the classes as well as a diverse range of variety for data.

### 4.4 Capturing Images of the Rice Kernels

The paddy images were captured in September 2023 using two microscope camera devices with the aim of creating a dataset useful for training AI models. In total, 100 kernels of each variety in 4 orientations have been captured to achieve 400 images from each type. All 14000 images are captured with two microscopic cameras with different zooms; the technical specifications are described in Table 3.

**Table 3:** Digital microscopic camera features.

Device	Image Resolution	Zoom
Digital Microscope	640×480p	1600X
Digital Microscope	640×480p	1000X

To ensure the quality of the images, a bright place with an abundance of light has been chosen. A tripod has been used to properly manage the angle of the camera. The session is conducted inside a closed room to avoid dust particles. With the help of a black cloth, the background was prepared to resist light reflection and provide complete focus on each kernel. To capture the prime features based on the hull of a paddy, the kernel of the paddy was photographed separately.

### 4.5 Validating the Images of the Dataset

To access the microscope camera device a designated software named “Pluggable Digital Viewer” is installed. The system autonomously generates images with a 640×480p resolution. Additionally, the captured images are saved in JPG format on the device. Pictures are then categorized into subfolders named with distinct rice species. Noisy, blurred, and irrelevant images are deleted. Ultimately, each of the 35 subfolders contained 400 RGB, microscopic, clear, and accurate paddy images that summed up to 14000 images. All the pictures, folders, and sub-folders are properly labeled and ready to be used by the machine learning model training.

### 4.6 Data Augmentation

Data augmentation is a crucial step in enhancing the diversity of image datasets for building robust machine learning and deep learning models. For this task, the Image Data Generator [9] is configured with various parameters. A rotation range adds random rotations within a specific degree range, whereas a shear range makes shear alterations. Pictures are reversed both vertically and horizontally. An augmented image of 640×480p is produced from the original image size. 1600 images per class have been prepared where each image utilizes augmentation to create a total of 4 additional images. To develop an enriched dataset for better model training, the augmentation samples comprise changes such as different angles, rotations, shears, and flips [7].

## Limitations

• Data collection did not include all existing varieties, creating a gap. Hence, the dataset may not present the full diversity of the cultivated paddy varieties.
• The imaging process was carefully controlled, but inconsistencies in lighting, angles, or other factors may exist across images. Although two microscopic cameras were used, this may limit the photography detail of the kernels.

## Ethics Statement

The ethical prerequisites for publishing in Data in Brief have been thoroughly reviewed and adhered to. It is hereby validated that the present study does not entail the participation of animal experimentation, human subjects, or the utilization of any data acquired from social media platforms.

## CRediT authorship contribution statement

**Md Tahsin:** Investigation, Data curation. **Muhammad Ibrahim:** Conceptualization, Writing – review & editing, Validation, Methodology. **Anika Tabassum Nafisa:** Data curation. **Maksura Binte Rabbani Nuha:** Data curation, Writing – original draft, Writing – review & editing. **Mehrab Islam Arnab:** Data curation, Writing – original draft, Writing – review & editing. **Md. Hasanul Ferdaus:** Methodology, Data curation. **Mohammad Manzurul Islam:** Writing – review & editing, Validation. **Mohammad Rifat Ahmmad Rashid:** Writing – review & editing, Validation. **Taskeed Jabid:** Project administration, Investigation. **Md. Sawkat Ali:** Conceptualization, Supervision, Project administration. **Nishat Tasnim Niloy:** Methodology, Writing – review & editing, Validation.

## Data Availability

Mendeley DataPaddyVarietyBD: Classifying Paddy Variations of Bangladesh with a Novel Image Dataset (Original data).
